# Adenosine Receptors Differentially Regulate the Expression of Regulators of G-Protein Signalling (RGS) 2, 3 and 4 in Astrocyte-Like Cells

**DOI:** 10.1371/journal.pone.0134934

**Published:** 2015-08-11

**Authors:** Till Nicolas Eusemann, Frank Willmroth, Bernd Fiebich, Knut Biber, Dietrich van Calker

**Affiliations:** Department of Psychiatry and Psychotherapy, University of Freiburg Medical Center, Freiburg, Germany; Temple University School of Medicine, UNITED STATES

## Abstract

The “regulators of g-protein signalling” (RGS) comprise a large family of proteins that limit by virtue of their GTPase accelerating protein domain the signal transduction of G-protein coupled receptors. RGS proteins have been implicated in various neuropsychiatric diseases such as schizophrenia, drug abuse, depression and anxiety and aggressive behaviour. Since conditions associated with a large increase of adenosine in the brain such as seizures or ischemia were reported to modify the expression of some RGS proteins we hypothesized that adenosine might regulate RGS expression in neural cells. We measured the expression of RGS-2,-3, and -4 in both transformed glia cells (human U373 MG astrocytoma cells) and in primary rat astrocyte cultures stimulated with adenosine agonists. Expression of RGS-2 mRNA as well as RGS2 protein was increased up to 30-fold by adenosine agonists in astrocytes. The order of potency of agonists and the blockade by the adenosine A2B-antagonist MRS1706 indicated that this effect was largely mediated by adenosine A2B receptors. However, a smaller effect was observed due to activation of adenosine A2A receptors. In astrocytoma cells adenosine agonists elicited an increase in RGS-2 expression solely mediated by A2B receptors. Expression of RGS-3 was inhibited by adenosine agonists in both astrocytoma cells and astrocytes. However while this effect was mediated by A2B receptors in astrocytoma cells it was mediated by A2A receptors in astrocytes as assessed by the order of potency of agonists and selective blockade by the specific antagonists MRS1706 and ZM241385 respectively. RGS-4 expression was inhibited in astrocytoma cells but enhanced in astrocytes by adenosine agonists.

## Introduction

The “regulators of G-protein signalling” (RGS) comprise a large family of proteins that act as very effective “off-switches” of receptor-activated cellular signal transduction [1–3]. RGS proteins have recently attracted much attention since one member of this family, RGS4, is considered as a candidate vulnerability marker for schizophrenia and may also play a role in bipolar disorder and Alzheimer’s disease [4–6]. Another RGS protein, RGS2, appears to play an important role in depression and anxiety [7,8] and aggressive behaviour [9–12] and maybe genetically involved in the biological susceptibility to suicide [13]. RGS proteins such as RGS2 and RGS4 are also important in the mechanism of action of drugs of abuse [14–16].

RGS proteins limit by virtue of their “GTPase accelerating protein” (GAP) activity the signal transduction of G-protein coupled receptors. G-proteins are trimeric proteins, which dissociate during receptor activation into the GTP-binding α-subunit exhibiting intrinsic GTPase activity and the dimeric β,γ-subunit. The GTPase activity of the active G-protein α-subunit, which terminates G-protein activation, is activated by the GAP-domain of RGS-proteins [3,17]. In addition to their GAP activity some RGS proteins appear to have other functions in the cell. Thus, e.g. a truncated form of RGS3 (RGS3T) directs apoptotic programs in cells [18], while another RGS3 variant with an extended N terminus,PDZ-RGS3, regulates reverse signalling of the neurotrophinEphrinB which is e.g. important in neural stem cell proliferation and positioning during neurogenesis [19–21]. Other functions of RGS proteins in the brain include regulation of neuronal cell differentiation and synaptic plasticity [22,23]. RGS proteins are thus considered as potential new drug targets for neuropsychiatric diseases [24].

Little is known about the regulation of cellular expression of RGS proteins. The expression of RGS2 and RGS4 in the striatum is differentially regulated by dopamine D1 and D2 receptors [25,26], but little is known about the potential effects of other neurotransmitters or neuromodulators. Since it was reported that the expression of some RGS mRNA’s in the brain was regulated by electroconvulsive treatment as well as seizures and ischemia [27–29], conditions that induce a large increase of adenosine in the brain [30–32], we hypothesized that adenosine might regulate RGS expression.

Adenosine’s numerous functions in the brain include e.g. the regulation of sleep and arousal as well as anticonvulsant and neuroprotective effects [30–32]. The four adenosine receptor subtypes, A_1_, A_2A_, A_2B_, and A_3_ all belong to the family of 7 transmembrane receptors that predominantly signal through activation of G-proteins. Adenosine receptors expressed on astrocytes regulate e.g. neuron-glia communication [33], neural resilience [34] and immune processes in the brain [35]. Variations in “gliotransmission” in the astrocyte network by virtue of altered signalling through glutamate, ATP and its metabolite adenosine are now considered to play a major role in neuropsychiatric diseases such as epilepsy and schizophrenia [36]. We report here that activation of adenosine A_2A_ and A_2B_ receptors differentially regulates the cellular expression of RGS2, RGS3 and RGS4 in astrocyte-like cells in culture.

## Materials and Methods

### Materials

Cyclopentyladenosine (CPA), 5’-(N-ethylcarboxamido)adenosine (NECA), and 8-Cyclopentyl-1,3-dipropylxanthine (DPCPX) were purchased from Sigma-Aldrich, Munich, Germany. 2-[4-(2-carboxyethyl)phenethylamino]-5′-*N*-ethylcarboxamidoadenosine (CGS-21680), 1-deoxy-1-(6-[([3-lodophenyl]methyl)-amino]-9H-purin-9-yl)-*N*-methyl-*β*-D-ribofuranuronamide (IB-MECA) and N-(4-Acetylphenyl)-2-[4-(2,3,6,7-tetrahydro-2,6-dioxo-1, 3-dipropyl-1H-purin-8-yl)phenoxy]acetamide (MRS 1706) were obtained from Tocris (Distributed by BIOZOL Diagnostica, Eching, Germany). Stock solutions (5 mM up to 100mM) were prepared in Ethanol (CPA) or DMSO (all other) and stored at –20°C. Further dilutions were carried out in distilled water.

### Cell culture

The human astrocytoma cell line U373 MG was obtained from the American Type Culture Collection (Rockville, USA) and was grown in MEM-Earle's medium (Invitrogen, Karlsruhe, Germany) containing 10% fetal calf serum (FCS) (Biochrom, Berlin, Germany), 2 mM L-glutamine (PAA, Cölbe, Germany), 1 mM sodium pyruvate, and 0,4% MEM non-essential amino acids (purchased from Invitrogen, Karlsruhe, Germany). Confluent monolayers were passaged routinely by trypsinization. Cells were plated for RNA isolation in 6-well dishes the day before treatment. Cultures were grown at 37°C in a 5% CO_2_ atmosphere.

Astrocyte cultures were established as described previously [37]. In brief, rat cortex was dissected under sterile conditions from newbornWistar rats (< 1d) killed by decapitation under ether anesthesiaas approved by the regional board of the Regierungspräsidium Freiburg, permit number X-13/06A. The brain tissue was gently dissociated by trituration in Dulbecco’s phosphate buffered saline and filtered through a cell strainer (70μm ø, Falcon) into Dulbecco’s modified Eagle`s medium (DMEM). After two washing steps (200xg for 10 min), cells were seeded into standard dishes (Falcon, 10cm diameter, 8x106 cells/dish). Cultures were maintained for up to 10 weeks in DMEM containing 10% foetal calf serum with 0,01% penicillin and 0,01% streptomycin in a humidified atmosphere (5% CO2) at 37°C. Culture medium was changed on the second day after preparation and every six days thereafter. For western blot, total RNA and mRNA preparation, cells were transferred to 6-well dishes (Falcon, 2x106 cells/dish). If not stated otherwise, cells were treated for 2h (U373) or 3h (rat astrocytes) with the respective agonists. Antagonists were added 30 minutes prior to the addition of agonists.

### RNA-Isolation, DNase-Treatment and reverse Transcription

Cells were lysed in guanidiniumisothiocyanate/mercaptoethanol (GTC) solution and total RNA was extracted by phenol extraction according to [38].

To prevent PCR signals derived from genomic DNA contaminations, the isolated RNA was treated with DNase (*DNA-free* Kit, Ambion Ltd., Huntingdon, UK). For reverse transcription 1 μg of RNA was incubated with 0,5 μg Oligo(dT) (Roche, Mannheim) for 5 min at 70°C and then transcribed with M-MLV Reverse Transcriptase (Promega, Heidelberg, Germany) according to the protocol of the supplier.

### Realtime PCR

Realtime PCR was applied to quantify human RGS2 cDNA [gi:142365756], human RGS3 cDNA [gi:62865652], human RGS4 cDNA [gi:38201693] and human Cyclophilin B cDNA [gi:44890060], rat RGS2 cDNA [gi:16758193], rat RGS3 cDNA [gi:18644717], rat RGS4 cDNA [gi:8394182] and rat Cyclophilin B cDNA [gi:11968125] using the Absolute QPCR SYBR Capillary mix (Abgene, Hamburg, Germany) and 300 nM of each primer and 1 μl sscDNA in a 20 μl reaction volume. Amplicons were designed using Vector NTI 10 (Invitrogen, Karlsruhe, Germany). The Primers used for human cDNA were as follows: The RGS2 amplicon consists of forward primer ATGTGCAAGGGTATTGAAGTTCTTATGAC,pos. 1105–1133, and reverse primer TTTTTGGCACTCATAACGGACACTG, pos. 1269–1245. The RGS3 amplicon consists of forward primer GACAACCTGCAGAGCGTCAC, pos. 1928–1947 and reverse primer GGTCAGAACGGAGAAAGCGA, pos. 2030–2011. The RGS4 amplicon consists of forward primer ATTGTACCCTTCTTGTCTCTCTGGCA, pos. 2524–2549 and reverse primer TCAGCGTGACCTTCCAGACCTATTA, pos. 2742–2718. The Cyclophilin B amplicon consists of forward primer AGGGCGGAGACTTCACCAG, pos. 477–495 and reverse primer GAAGCGCTCACCGTAGATGC, pos 538–519.

The Primers used for rat cDNA were: The RGS2 amplicon consists of forward primer CCTTTCCCTGAACTAGTCCATGTTACC, pos. 1001–1027 and reverse primer CACACCATTTAACAATGCAAACACG, pos. 1094–1070. The RGS3 amplicon consists of forward primer GGCGGAATGAATCTCCTGG, pos. 2475–2493 and reverse primer TTCCTCGGAGGTAGGCTTGA, pos. 2554–2535. The RGS4 amplicon consists of forward primer AAATTCCATGCATGAGCACCATTG, pos. 2401–2424 and reverse primer GACACGACTGTGGACTTGTGTATTACAAA, pos. 2569–2597. The Cyclophilin B amplicon consists of forward primer CATGATCCAGGGTGGAGACTTC, pos. 289–319 and reverse primer AGTGCTTCAGCTTGAAGTTCTCATC, pos. 395–371. All primers were synthesized by MWG, Ebersberg, Germany.

Realtime PCR was performed on a Roche Light Cycler 1.5 with 15 min 95°C and 40 cycles of 15 sec 95°C and 1 min at 60°C. Data given are arbitrary units (ratio of RGS2, -3, -4 mRNA and CycB mRNA). Data collection and processing was performed with Roche Light Cycler Software 3.0. To quantify the relative expression of RGS2, -3, -4 or Cyclophilin-B, an arbitrary cDNA containing reasonable amounts of target cDNA was serially diluted for the realtime PCR to obtain a standard curve. The number of cycles needed to obtain a certain fluorescence light level (cycle treshold, Ct) from an unknown probe was recalculated on the basis of the standard curve into the arbitrary unit “nanoliter cDNA”. Finally, expression of RGS2, -3 and -4 was normalised to the endogenous control gene Cyclophilin-B by dividing arbitrary units of RGS2, -3 or -4 by arbitrary units of Cyclophilin-B.

### Western blot analysis

Astrocyte cultures grown on 6-well dishes were washed once with ice-cold PBS and lysed in 300μl of buffer containing 25 mM HEPES (pH 7.5), 150 mMNaCl, 10% glycerol, 10 mM MgCl_2_, 1% triton x-100, and protease inhibitors (1 mg/ml leupeptin, 1 mg/ml aprotinin, 1 mMphenylmethylsulfonyl fluoride). The lysates were cleared from insoluble material by centrifugation at 12,000 g for 15 min. The samples were boiled in Laemmli buffer for 5 min, subjected to electrophoresis, and analyzed by Western blotting with 0.1 μg/ml chicken anti-RGS2 antibody (GenWay Biotech, San Diego, California, USA) followed by 0.15 μg/ml horseradish peroxidase-conjugated rabbit anti-chicken antibody (Abcam, Cambridge, UK). Equal loading was confirmed with 0.1 μg/ml horseradish peroxidase-conjugated goat anti-GAPDH antibody (Santa Cruz Biotechnology, Heidelberg, Germany).

### Statistics

Statistics and graphics were done with GraphPad Prism version 5.01. All data are given as means ± SEM. The statistical significance of differences between mean values was assessed using Student's *t*-test. Differences were regarded as statistically significant for *P*<0.05.

## Results

### RGS mRNA expression in U373 MG astrocytoma cells

**Fig 1:**
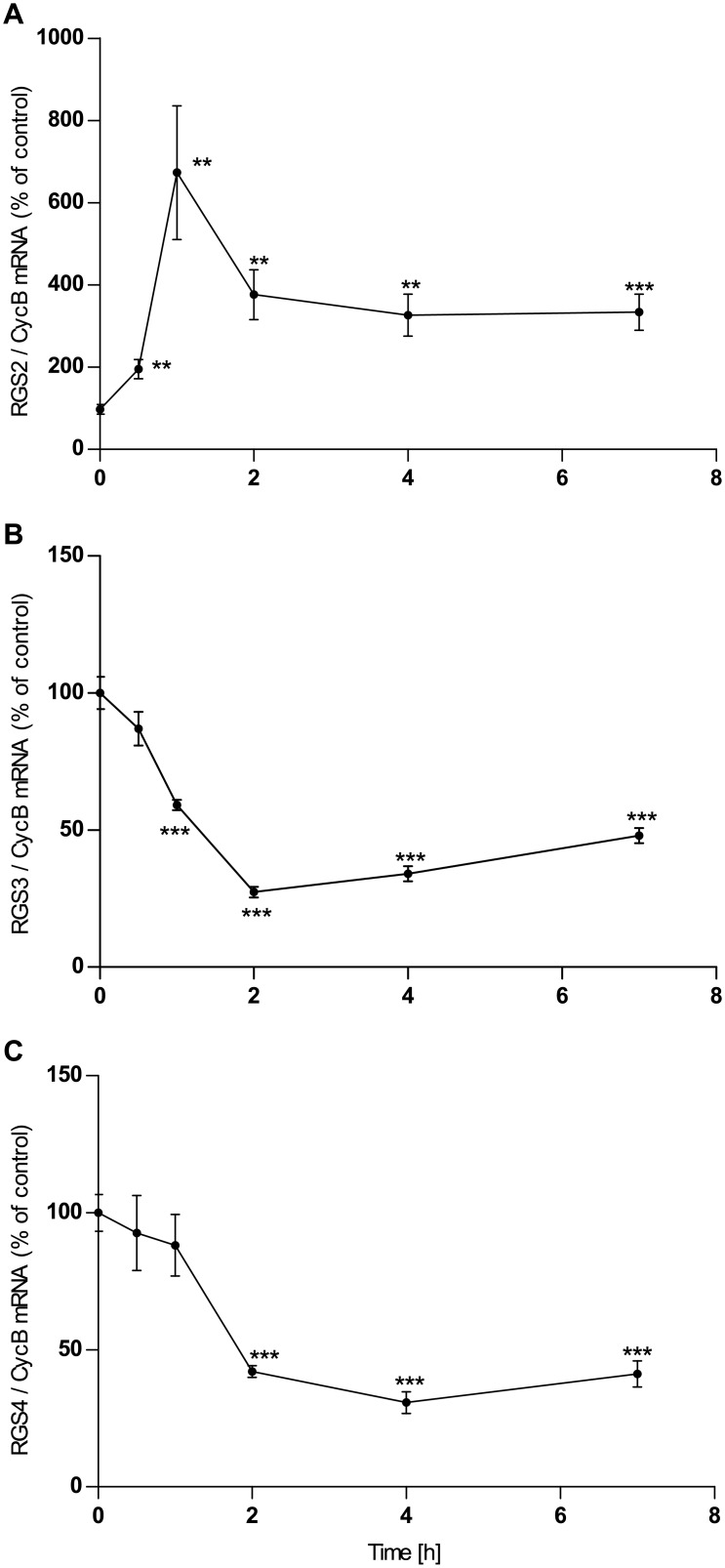
Time course of the effect of NECA (1μM) on the expression of mRNA of RGS2 (A), RGS3 (B) and RGS4 (C) in human astrocytoma cells U373 MG. Cells were cultured, incubated with NECA and assayed for mRNA expression by quantitative realtime PCR as described in Materials and Methods. Data given are means of five replica culture dishes ±SEM. A repetition of the experiment gave a similar result. ** p<0.01, *** p<0.001, student’s t-test, two-tailed.

**Fig 2:**
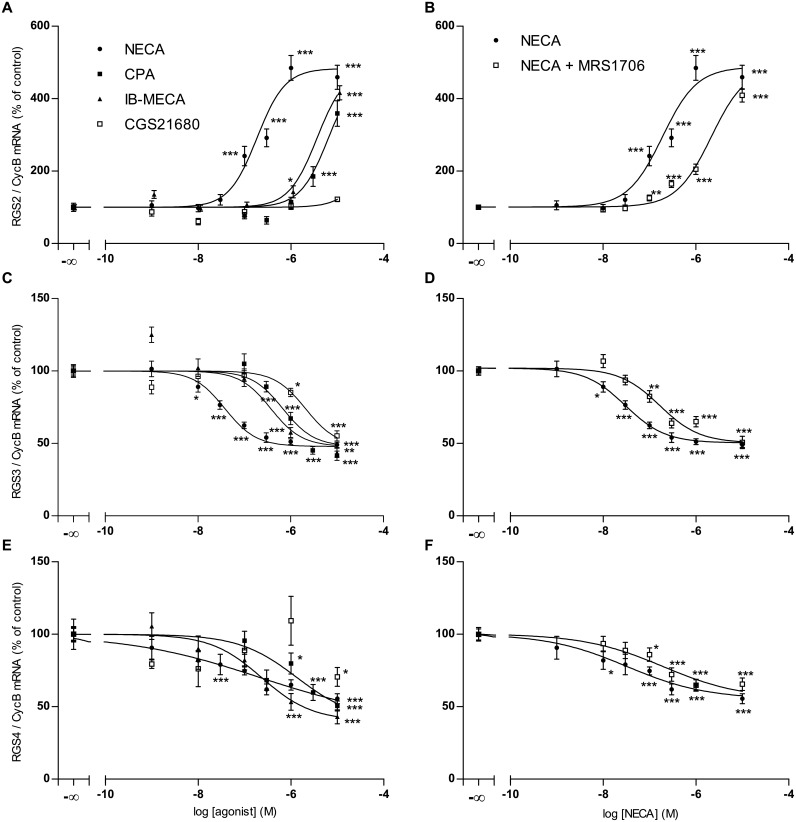
Effects of adenosine agonists and the adenosine A2B receptor antagonist MRS1706 (500 nM) on the expression of mRNA of RGS2 (A,B), RGS3 (C,D) and RGS4 (E,F) in human astrocytoma cells U373 MG. Cells were cultured, incubated with agonists/antagonists and assayed for mRNA expression by quantitative realtime PCR as described in Materials and Methods. Data given are means of 5 experiments (NECA, NECA/MRS1706) or 2 experiments (CPA, IB-MECA, CGS21680) each done in five replica culture dishes.* p<0.05, ** p<0.01, *** p<0.001, student’s t-test, two-tailed.

Incubation with the non-selective adenosine receptor agonist NECA leads to up-regulation of expression of RGS2 mRNA and down-regulation of RGS3 and RGS4 in U373 astrocytoma cells. Maximum effects are reached after 2 hours of incubation (1 h in the case of RGS2) and persist up to at least 5 hours (Fig 1A–1C). The closer pharmacological examination revealed the following:

**RGS2**. NECA is at least one order of magnitude more potent in eliciting the increase in RGS2 mRNA than the specific adenosine A1-agonist CPA and the A3-agonist IB-MECA, indicating that the effect is mediated by A2-receptors. The specific A2A-agonist CGS21680 is even less potent than CPA and IB-MECA indicating that the effect is mediated solely by the A2B subtype (Fig 2A). Accordingly, the effect of NECA is antagonized by the specific and selective A2B-antagonist MRS1706 (Fig 2B). The specific A1-Antagonist DPCPX (0.1 μM) did not antagonize the effects of NECA on RGS2 expression (results not shown).
**RGS3**. The order of potency of adenosine agonists in the down-regulation of RGS3 expression in astrocytoma cells is NECA>CPA/IB-MECA> CGS21680 indicating that the effect is due to activation of A2B-receptors (Fig 2C). Accordingly, the effect of NECA is antagonized by the specific and selective A2B-antagonist MRS1706 (Fig 2D) The specific A1-Antagonist DPCPX (0.1 μM) did not antagonize the effects of NECA on RGS3 expression (results not shown).
**RGS4**. In the case of RGS4 the concentration response curves in astrocytoma cells are much shallower and the difference in potency between the various agonists is less pronounced,except for CGS21680, which has no clear effect (Fig 2E). The specific A1-Antagonist DPCPX (0.1 μM) did not antagonize the effects of NECA on RGS4 expression (results not shown), the specific and selective A2B-antagonist MRS1706 had only a minor influence on the effect of NECA on RGS4 expression (Fig 2F).

### RGS mRNA expression in cultured astrocytes

**Fig 3:**
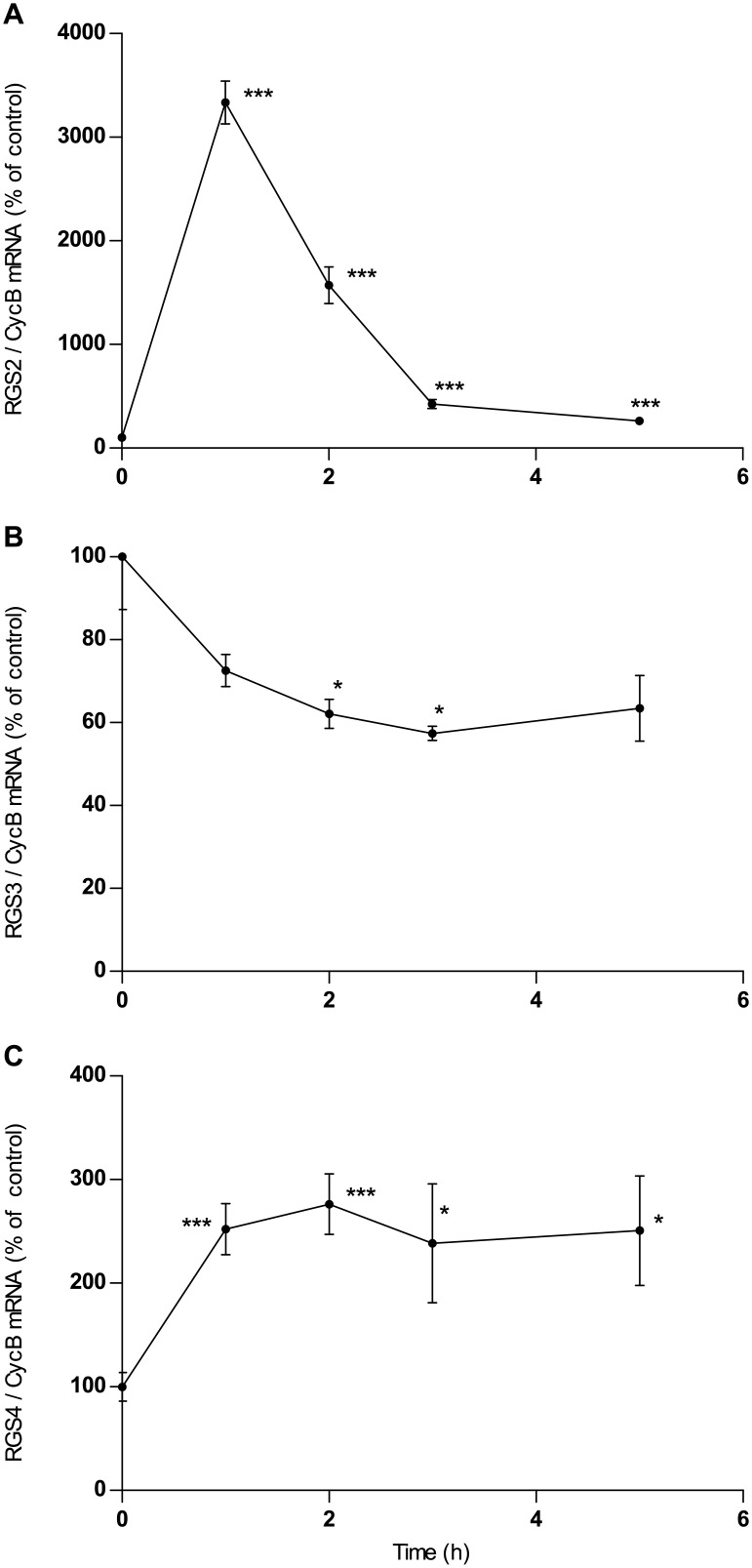
Time course of the effect of NECA (1μM) on the expression of mRNA of RGS2 (A), RGS3 (B) and RGS4 (C) in rat astrocytes. Cells were cultured, incubated with NECA and assayed for mRNA expression by quantitative realtime PCR as described in Materials and Methods. Data given in A and B are means of five replica culture dishes ±SEM. Two repetitions of the experiment gave similar results. Data given in C are means of 3 different experiments done in four replica culture dishes. *p<0.05, ** p<0.01, *** p<0.001, student’s t-test, two-tailed.

**Fig 4:**
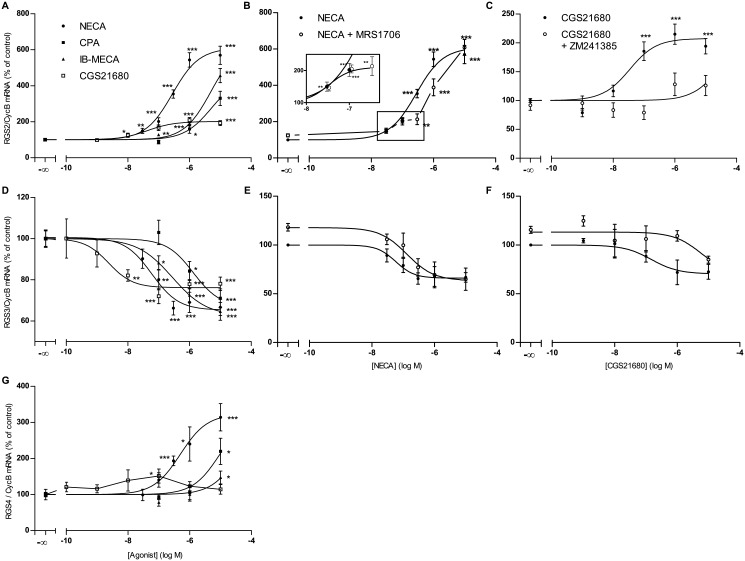
Effects of adenosine agonists and the adenosine A2A and A2B receptor antagonists, MRS1706 (500nM) and ZM241385 (1μM), on the expression of mRNA of RGS2 (A,B,C), RGS3 (D,E,F) and RGS4 (G) in rat astrocytes. Cells were cultured, incubated with agonists/antagonists and assayed for mRNA expression by quantitative realtime PCR as described in Materials and Methods. Data given are means of 4 (NECA, CGS21680), 3 (CPA, IB-MECA, CGS21680/ZM241385) or 5 (NECA/MRS1706) experiments each done with 4 replica culture dishes. * p<0.05, ** p<0.01, *** p<0.001, student’s t-test, two-tailed.

Incubation with the non-selective adenosine receptor agonist NECA leads to up-regulation of expression of RGS2 mRNA and down-regulation of RGS3 in astrocytes (Fig 3A and 3B), similar to its effects in astrocytoma cells. However, while RGS4 is down-regulated by NECA in astrocytoma cells (Fig 1C) it is up-regulated in astrocytes (Fig 3C). The closer pharmacological examination revealed the following:

**RGS2**. Also in astrocytes NECA is at least one order of magnitude more potent in eliciting the increase in RGS2 mRNA than the specific adenosine A1-agonist CPA and the A3-agonist IB-MECA and the effect of CGS21680 is much less pronounced than that of NECA indicating that also in astrocytes the effect is mediated largely by A2B-receptors (Fig 4A). However, even at low concentrations CGS21680 does elicit a 2-fold increase in RGS2 expression indicating that a minor component of the total increase in RGS2 expression is due to activation of A2A-receptors. The A2B-antagonist MRS1706 potently antagonized the effect of NECA on RGS2 expression (Fig 4B). However, this is only true for higher concentrations of NECA. Concentrations of NECA ≤100nM still elicited a 2-fold increase in RGS2 mRNA that was not blunted by the presence of MRS1706 (Fig 4B insert), compatible with mediation by A2A-receptors. Consistent with a role of A2A-receptors, the increase of RGS2 expression in astrocytes induced by CGS21680 is blunted by the A2A-antagonist ZM241385 (Fig 4C). The specific A1-Antagonist DPCPX did not antagonize the effects of NECA on RGS2 expression (results not shown). In summary, our data suggest that activation of A2A as well as A2B receptors leads to an increase in RGS2 expression, with the increase mediated by A2B receptors being about 3 times greater.
**RGS3**. The order of potency of adenosine agonists in the down-regulation of RGS3 in astrocytes is CGS21680>NECA>IB-MECA>CPA (Fig 4D), indicating mediation by A2A-receptors (in contrast to astrocytoma cells, where the effect is mediated by A2B receptors, see above). The inhibition of RGS3 expression in astrocytes induced by NECA is not antagonized by MRS1706 (Fig 4E), whereas the effect of CGS21680 on RGS3 expression is antagonized by the A2A antagonist ZM241385 (Fig 4F), both consistent with mediation by A2A-receptors. The specific A1-Antagonist DPCPX did not antagonize the effects of NECA on RGS3 expression (results not shown).
**RGS4**. In astrocytes the expression of RGS4 is extremely low and at the border of sensitivity of the assay and is, in contrast to astrocytoma cells, not down-regulated but rather up-regulated by adenosine agonists (Fig 4G). NECA is the most potent agonist while effects of the other agonists are much less pronounced indicating a predominant role of A2B-receptors. The specific A1-Antagonist DPCPX did not antagonize the effects of NECA on RGS4 expression (results not shown). The very low expression of RGS4 in astrocytes precluded any detailed further analysis of the effects of adenosine receptor antagonists on RGS4 expression.

### Effects of NECA on RGS protein expression in astrocytes

**Fig 5:**
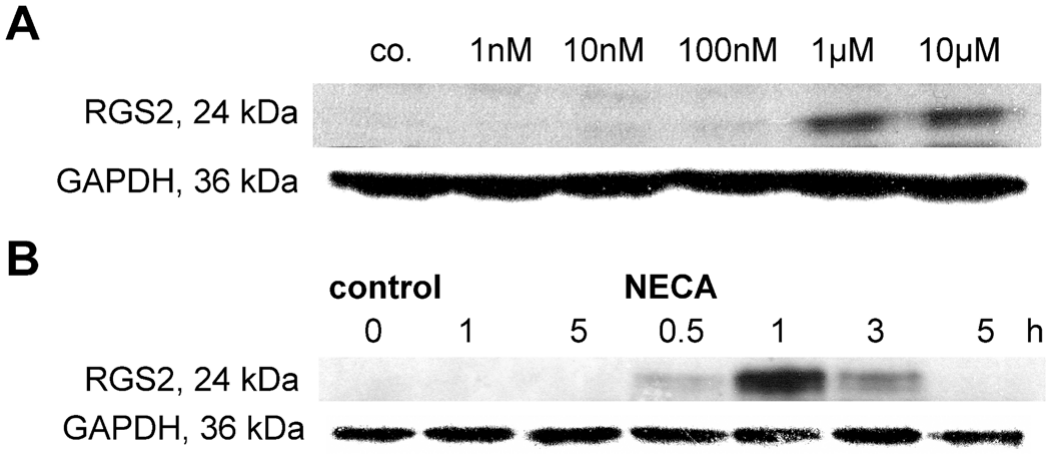
Concentration-response relationship (A) and time course (B) of the effect of NECA on expression of RGS2 protein in cultured rat astrocytes. Cells were cultured, incubated with NECA (concentrations as indicated in A; 1 μM in B) and assayed for protein expression by western blotting as described in Materials and Methods. Shown are the results of one typical experiment, one repetition gave similar results.

The increase in RGS2 mRNA expression in astrocytes is accompanied by an increase of RGS2 protein expression as shown by western blot. NECA elicits a concentration-dependent increase in RGS2 protein (Fig 5A) which is maximally pronounced at 1 h and has already disappeared at 5 h of incubation (Fig 5B). In our hands the limited specificity of the commercially available antibodies for RGS3 and RGS4 precluded a reliable analysis of RGS3 and RGS4 protein by western blot. Similar problems have been encountered by others [3].

## Discussion

### Adenosinergic regulation of RGS proteins in U373 MG human astrocytoma cells

The present work shows that in U373 astrocytoma cells adenosine agonists up-regulate RGS2 mRNA expression and down-regulate expression of RGS3 and RGS4. The pharmacological characterization clearly suggests that the effects on RGS2 and RGS3 expression are mediated by adenosine A_2B_-receptors.

However, the effects of adenosine agonists on RGS4 expression are much less clear. Neither A_3_, nor A_1_, nor A_2A_-receptors alone can be responsible for this effect, since in this case the respective agonists IB-MECA, CPA and CGS21680 should be much more potent than NECA. On the other hand, NECA should be much more potent than IB-MECA or CPA, if the effect was mediated merely by A_2B_-receptors, as seen in the cases of RGS2 and RGS3 (Fig 2A and 2C). Furthermore, the A_2B_-antagonist MRS1706 does not appreciably alter the actions of NECA on RGS4 expression. Together with the shallow concentration–response curves these findings suggest that combined action of more than one adenosine receptor subtype might be involved in the regulation of RGS4 expression [39]. Further work will be needed to identify the mechanism of regulation of RGS4 expression in U373 cells.

### Adenosinergic regulation of RGS proteins in cultured rat astrocytes

The effects of adenosine agonists on RGS-2, -3, -4 expression in cultured astrocytes differ in several aspects from those in U373MG astrocytoma cells.

First, while similar to U373 cells, also in astrocytes the increase in RGS2 evoked by adenosine agonists is predominantly mediated by adenosine A_2B_-receptors, a small component of this effect is due to adenosine A_2A_-receptor activation. This is concluded from the following findings:1) NECA elicits a biphasic concentration response curve in the presence of theA_2B_-antagonist MRS1706, which antagonizes the effects of higher but not of lower concentrations of NECA (Fig 4B);2) low concentrations of the specific A_2A_-agonist CGS21680 upregulate RGS2 expression to a limited extent (Fig 4A and 4C), an effect antagonized by the specific A_2A_-antagonist ZM 241385, (Fig 4C).

Second, in astrocytes the inhibition by adenosine agonists of RGS3 expression is mediated by A_2A_-receptors and not by A_2B_-receptors as in U373 cells as concluded from the effects of adenosine agonists and antagonists (Fig 4). Third, in contrast to U373 astrocytoma cells, RGS4 is not downregulated but rather upregulated by adenosine agonists in astrocytes. This effect is most likely mediated by A_2B_-receptors (Fig 4F). However, expression of RGS4 in astrocytes was much lower than in U373MG astrocytoma cells precluding a reliable further assessment of the receptor type involved.

These differences in baseline expression and regulation of RGS proteins in (human) astrocytoma cells as compared to (rat) astrocytes may be due to species differences. Another scenario is that altered expression regulation patterns in astrocytoma cells are related to their transformation to tumour cells. Indeed, it was found that in U373MG as well as primary glioblastoma cells both adhesion and migration, possible promotors of malignancy, were enhanced in clones exhibiting an increased expression of RGS3 and -4. [40].

### Limitations of the present study

We have demonstrated by western blot that the increase in RGS2 mRNA evoked by adenosine agonists in astrocytes results in a concomitant increase of RGS2 protein. Demonstration of regulation of protein expression was not possible in the cases of RGS3 and RGS4. In our hands the commercially available antibodies did not allow reliable analysis of RGS3 and -4 proteins in western blots (results not shown). Further work with newly developed more specific antibodies is necessary to clarify whether or not the regulation of RGS3 and -4 mRNA by adenosine leads to the expected changes in protein expression.

Next, the identification of adenosine receptor subtypes involved is so far based on pharmacological tools. Confirmation of these findings in mice in future studies will allow the use of A_2A_- and A_2B_-receptor knockout mice to verify these pharmacological data [41,42]. Further studies will also analyze functional readouts of altered RGS function such as intracellular calcium signalling. With these caveats in mind it is tempting to speculate about the potential physiological and pathophysiological implications ofadenosinergic regulation of RGS expression in astrocytes.

### Possible consequences of regulation of RGS2,-3 and -4 in astrocytes

Astrocytes express receptors for a large number of neurotransmitters many of which are coupled to G-proteins[43]. By virtue of several of these receptors astrocytes respond to the synaptic release of various neurotransmitters, such as glutamate, noradrenaline and acetylcholine with transient, often repetitive elevations in intracellular calcium. Moreover, astrocytes can discriminate signals from different synapses and integrate these in a nonlinear fashion [44]. Astrocytes activated in that fashion can then release gliotransmitters such as glutamate, D-serine and ATP, that may eventually modulate signalling in neurons in areas remote from the initial stimulus [33,45], either by contact of the astrocyte’s processes to other synapses, or by propagation of calcium elevations to adjacent astrocytes via gap junctions and/or the release of ATP. The increase in [Ca^2+^]_i_ primarily depends on activation of receptors linked via G_q/11_–proteins to production of inositol 1,4,5-trisphosphate and subsequent release of Ca^2+^ from intracellular stores. In as much as intracellular signalling of these receptors is regulated by the GAP activity of particular RGS proteins, up- or down-regulation of RGS by adenosine as reported here should, respectively, desensitize or sensitize signalling through these receptors and thus modulate Ca^2+^ signalling and its propagation through the astrocytic network. Of note, the [Ca^2+^]_i_ oscillations often observed after activation of Gα_q/11_-protein coupled receptors require the action of RGS proteins [3,46] and may thus be modulated by changes in their expression. RGS2, -3 and -4 can all terminate signal transduction through G_q/11_-proteins and thus limit Ca^2+^-signalling. However, recent data have provided strong evidence that RGS proteins display remarkable selectivity towards specific receptors and G-proteins in their regulation of G-protein-mediated physiological events [47,48]. Thus, while RGS2, -3 and -4 all inhibit Gα_q/11_-proteins, they appear to differ in their ability to inhibit Gα_i_-proteins [49,50]. Thus, the adenosine-induced up-regulation of RGS2 (and perhaps RGS4) in astrocytes might impact other receptors and/or signal transducing systems than the down-regulation of RGS3.

As demonstrated in the present work, the expression of RGS2, -3 and -4 in astrocytes is regulated by two types of adenosine receptors, A_2B_ and A_2A_ receptors. A_2B_ receptors are low-affinity receptors activated only by high concentrations of adenosine that occur in pathological conditions such as seizures or hypoxia, while A_2A_ receptors are activated at physiological adenosine concentrations in the brain [31].

During physiological neuro- and gliotransmissionadenosine is releaseddepending on neuronal activity: first, ATP is released from astrocytes by virtue of SNARE-dependent exocytosisor by diffusion through CX43 hemichannelsand is subsequentlymetabolised to adenosine [51, 52]. Second, adenosine is also directly released from neurons via equilibrative nucleoside transporter proteins (ENTs) [53]andmediates feedback inhibition of excitatory activityduring prolonged activity[54]. It is removed from the extracellular space by reuptake into astrocytes and subsequent intracellular phosphorylation by adenosine kinase(for review see [30]). Under these physiological conditions, adenosine acting primarilyonA_2A_ receptors likely induces downregulation of RGS3 and limited upregulation of RGS2 in astrocytes.

On the other hand, pathological conditions such as ischemia lead to a large increase inextracellular adenosine by induction of CX43hemichannels in astrocytes and thus enhanced efflux of ATP subsequently broken down to adenosine [52]. Thismay well result in activation of A_2B_ receptors and thus a large induction of RGS2 expressionlikely outweighing changes in RGS3 expression. Seizures and ischemiaenhance the Ca^2+^ excitability of astrocytesfor prolonged periods of time that outlast the duration of the seizureitself for at least 3 days [55,56] due to persistent pathological activation of metabotropic glutamate and GABA_B_ receptors. Under these conditions the increase in RGS2 expression evoked by high concentrations of adenosine might serve to limit the extent of astrocyteCa^2+^ excitability by virtue of inhibiting signal transduction through Gα_q/11_-coupled receptors.

Adenosine originating from astrocyte-derived ATP has been found to play an important role in the regulation of both synaptic networks [57] and neuronal activity in larger neuronal networks regulating activities such as sleep and cognition [58,59]. Indeed cognitive consequences of sleep deprivation were absent in mice with distorted ATP/adenosine release properties [58]. Our data indicate that adenosine might also act back on astrocytes in an autocrine fashion to modulate their sensitivity to both neuro- and gliotransmitters.

In conclusion, we have provided evidence that expression of RGS2, -3, and -4 mRNA in astrocyte-like cells is differentially regulated by adenosine. Thus, adenosinergic mechanisms should be considered as a potential cause of alterations in RGS expression in the brain under various physiological and pathological conditions.

## Supporting Information

S1 Fig DPCPX (100nM) did not alter the effect of NECA (1μM) on RGS2- and RGS3 mRNA expression in rat astrocytes. (PDF)

S1 Table Aggregated experimental data of RGS2 mRNA expression in rat astrocytes incubated with NECA, CPA, CGS21680, IB-MECA (A-D); NECA in the presence of 1μM MRS1706 (E); CGS21680 in the presence of 1μM ZM241385 (F); and a fixed concentration of NECA at different durations of incubation (G). Data are normalised so that means of control data equal 100%.
(XLSX)

S2 Table Aggregated experimental data of RGS3 mRNA expression in rat astrocytes incubated with NECA, CPA, CGS21680, IB-MECA (A-D); NECA in the presence of 1μM MRS1706 (E); CGS21680 in the presence of 1μM ZM241385 (F); and a fixed concentration of NECA at different durations of incubation (G). Data are normalised so that means of control data equal 100%.
(XLSX)

S3 Table Aggregated experimental data of RGS4mRNA expression in rat astrocytes incubated with NECA, CPA, CGS21680, IB-MECA (A-D); NECA in the presence of 1μM MRS1706 (E); and a fixed concentration of NECA at different durations of incubation (F). Data are normalised so that means of control data equal 100%.
(XLSX)

S4 Table Aggregated experimental data of RGS2 mRNA expression in U373 MG human astrocytoma cells incubated with NECA, CPA, CGS21680, IB-MECA (A-D); and NECA in the presence of 1μM MRS1706 (E); Data are normalised so that means of control data equal 100%. (XLSX)

S5 Table Aggregated experimental data of RGS3 mRNA expression in U373 MG human astrocytoma cells incubated with NECA, CPA, CGS21680, IB-MECA (A-D); and NECA in the presence of 1μM MRS1706 (E); Data are normalised so that means of control data equal 100%. (XLSX)

S6 Table Aggregated experimental data of RGS4 mRNA expression in U373 MG human astrocytoma cells incubated with NECA, CPA, CGS21680, IB-MECA (A-D); and NECA in the presence of 1μM MRS1706 (E); Data are normalised so that means of control data equal 100%. (XLSX)
