# Supplementary figures and images for: Adenosine Receptors Differentially Regulate the Expression of Regulators of G-Protein Signalling (RGS) 2, 3 and 4 in Astrocyte-Like Cells
Source: PLoS One. 2015 Aug 11;10(8):e0134934. doi: 10.1371/journal.pone.0134934 (PMC4532427; doi:10.1371/journal.pone.0134934)

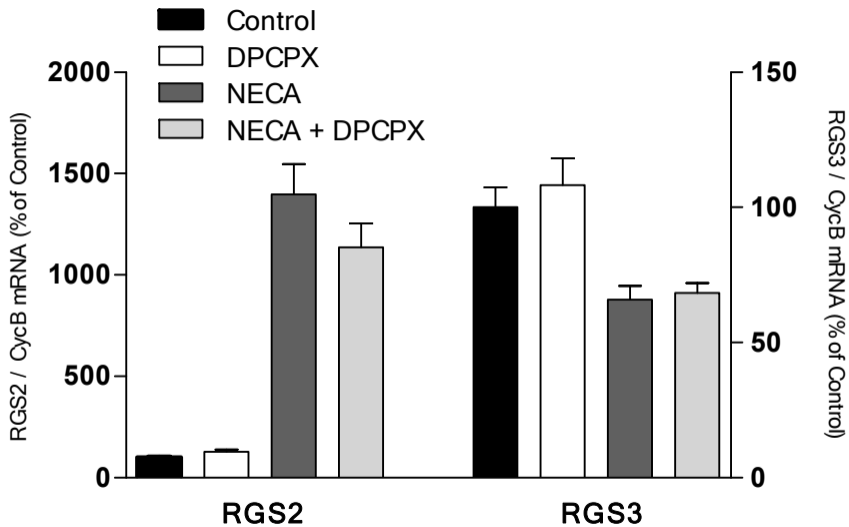

Supplement: S1 Fig — (PDF) [file pone.0134934.s001.pdf]
